# Supplementary material for: Heading choices of flying Drosophila under changing angles of polarized light
Source: Sci Rep. 2019 Nov 14;9:16773. doi: 10.1038/s41598-019-53330-y (PMC6856357; doi:10.1038/s41598-019-53330-y)

Heading choices of flying *Drosophila* under changing angles of polarized light

Thomas F. Mathejczyk and Mathias F. Wernet*

Freie Universität Berlin
Fachbereich Biologie, Chemie und Pharmazie
Institut für Biologie – Neurobiologie
Königin-Luise Strasse 1-3
14195 Berlin, Germany

*: Corresponding author
e-mail: mathias.wernet@fu-berlin.de
phone: +49-30-838-60115

Supplemental Material

Supplemental Figure S1: Setup overview

A. Photo of two flight simulators within a temperature- and humidity-controlled arena, one with a green (left) and one with a UV stimulus LED (right). (1) Collimated stimulus LED; (2) rotatable filter cassette holder; (3) upper magnet; (4) movable cylinder containing white LEDs; (5) humidifier. **B.** Same as A, but with the humidifier removed, revealing a heating plate and two fans in the back for controlling the arena temperature. **C.** Visualization of the polarized stimulus light path. For 3D printing instructions and a detailed description of the step-by-step assembly process, see https://doi.org/10.1101/527945 and www.flygen.org/skylight-navigation.

Supplemental Figure S2: Stimulus properties within the arena

A. Characterization of the optical spectrum of the LED light sources used (UV and green stimulus LEDs (Mightex, see materials and methods)), as well as white LED strips for back-illumination of the white cylinder surrounding the tethered fly (see below). **B.** Polarimetric characterization of stimuli presented dorsally to the flies within the apparatus. Top: UV stimulus, 365 nm (from left to right: (i) intensity, as well as the two filter/diffuser orientations shown in Figure 1B: (ii) polarized (center) and (iii) unpolarized (right)). False color legend indicating the degree of polarization (blue: unpolarized; red: fully polarized). Bottom: Same analysis for the linearly polarized green stimulus (510 nm). **C.** Detailed view of a tethered fly within the setup. Only light passing through the polarizer at incident angles lower than 30° (violet) is hitting the fly's eye directly, thereby minimizing polarizer transmission artefacts. $\alpha = 17°$. $\beta < 30°$. Legend: (1) Sheet polarizer; (2) bar magnet; (3) tethered fly; (4) ring magnet; (5) backlit matt white cylinder wall to prevent intensity artefacts due to linearly polarized light (as discussed in ref. 15 (white LED strips are symbolized as yellow circles); (6) matt white bottom plate with hole in the middle, through which the fly is filmed and air puffs can be delivered.

Supplemental Figure S3: Test for behavioral impairment

Bar plots depicting the sum of rotational motion for all tested flies per 5 min trial (positive = clockwise, negative = counter-clockwise), showing that the flies‘ ability to rotate around the yaw axis was not impaired, for any of the experimental conditions tested.

Supplemental Figure S4: Flies are able to keep a chosen heading relative to the evector even after a 5min break

A-C. Scatter plots showing the chosen preferred e-vector over consecutive trials UVPol1/UVPol2, UVPol1/UVUnpol1 and UVPol1/GRPol1, respectively, for all flies tested. Shading of points

indicates percentage of time each fly spent following the e-vector rotation in the first trial. Under polarized UV conditions flies that spent the most time following the e-vector rotation in the first trial are better at keeping this chosen angle in a second trial (indicated by clustering of dark points around the diagonal black line) compared to unpolarized UV and polarized green light, where this effect seems much weaker. **A‘-C‘.** In order to assess whether a preferred e-vector might be chosen randomly in a second trial, the mean angular difference between the preferred e-vectors chosen in two consecutive trials that was measured (black arrows) was compared to the distribution of the mean angular differences between the measured preferred e-vectors chosen in the first trial and sets of randomized e-vectors (shuffled 10000 times, blue distribution). Under polarized light the measured mean differences between the preferred e-vectors chosen in two consecutive trials show lower probabilities of occurring randomly compared to flies flying under unpolarized light. N = A/A‘:42; B/B‘:20; C/C‘:35.

Supplemental Figure S1

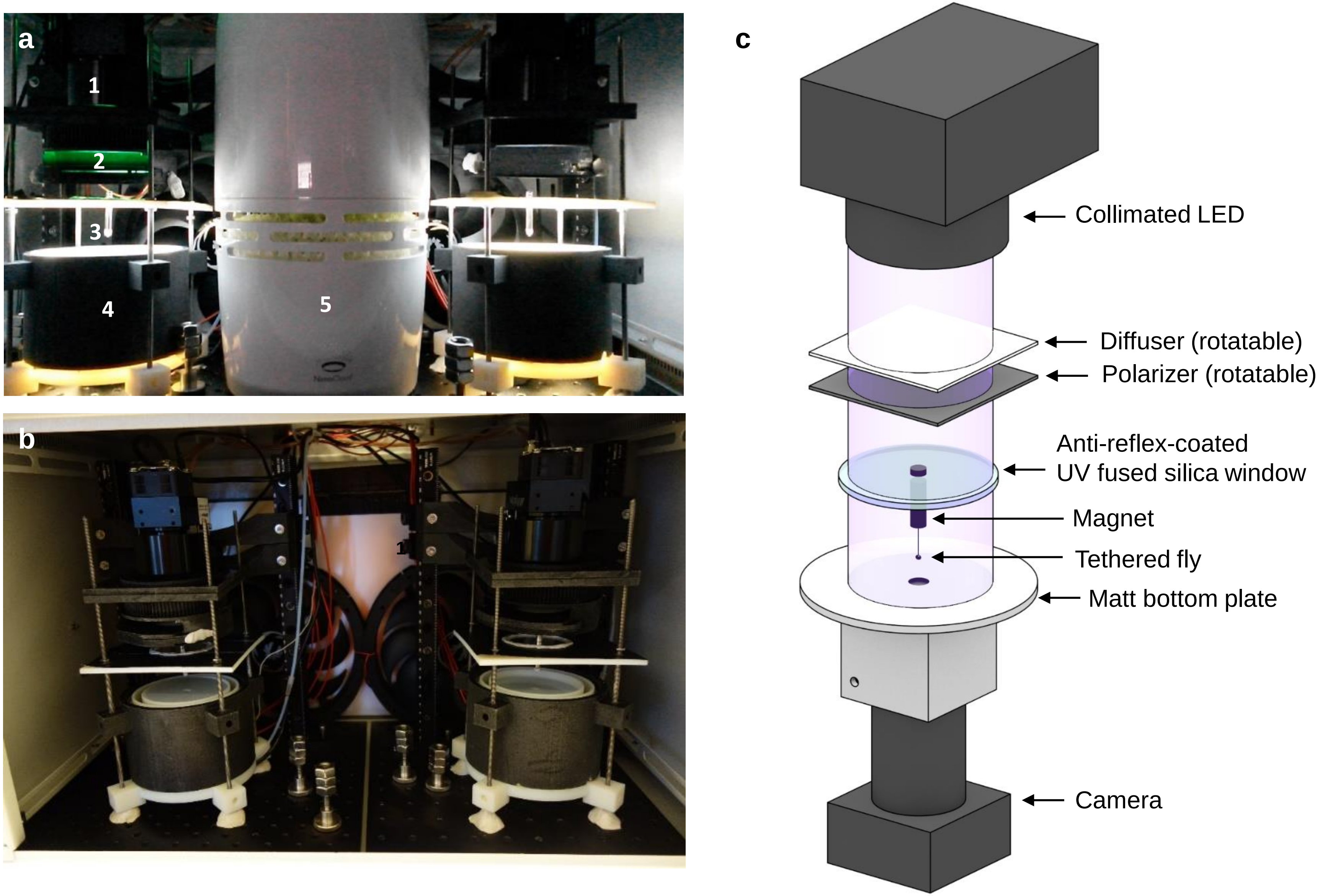

Supplemental Figure S2

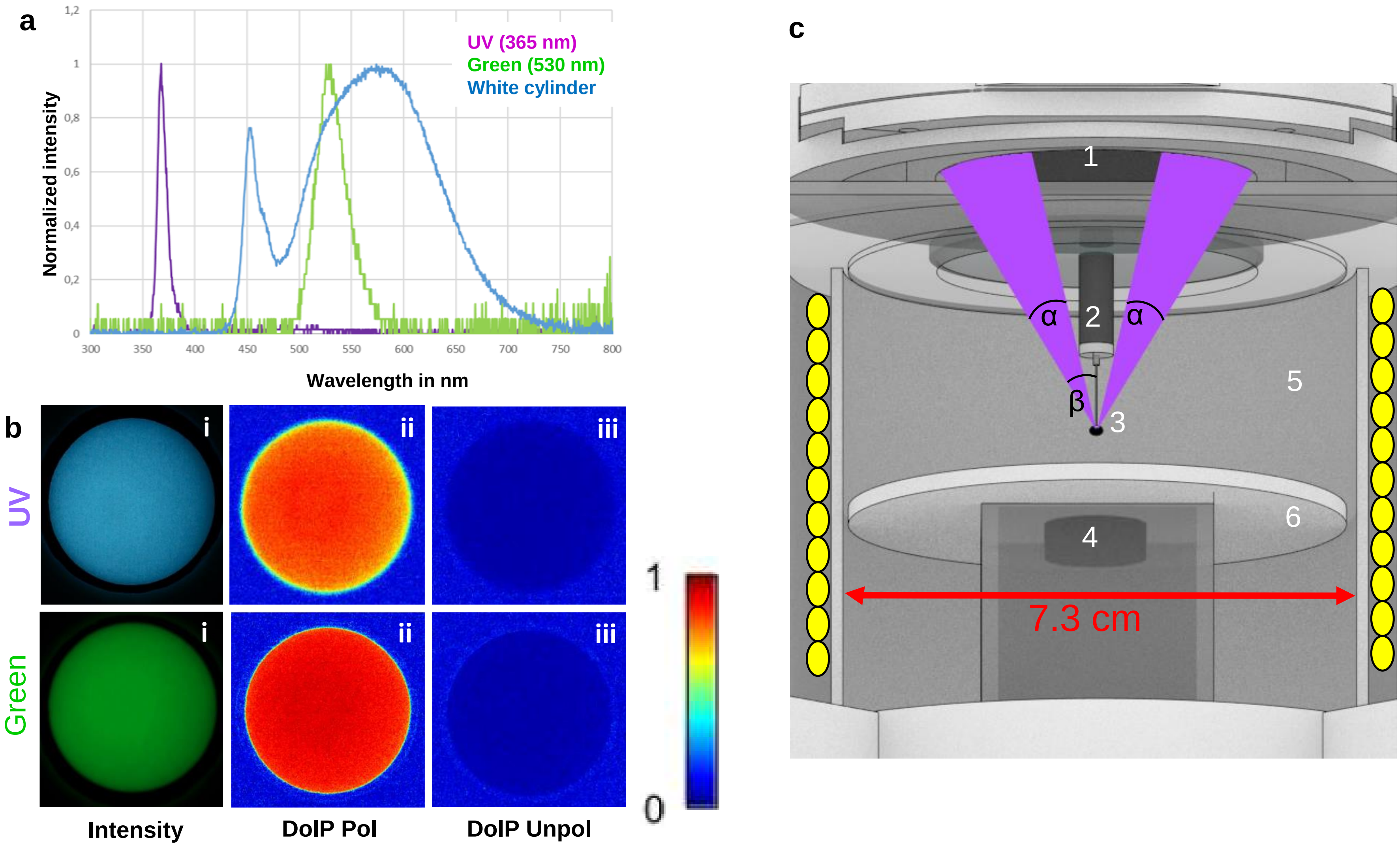

Supplemental Figure S3

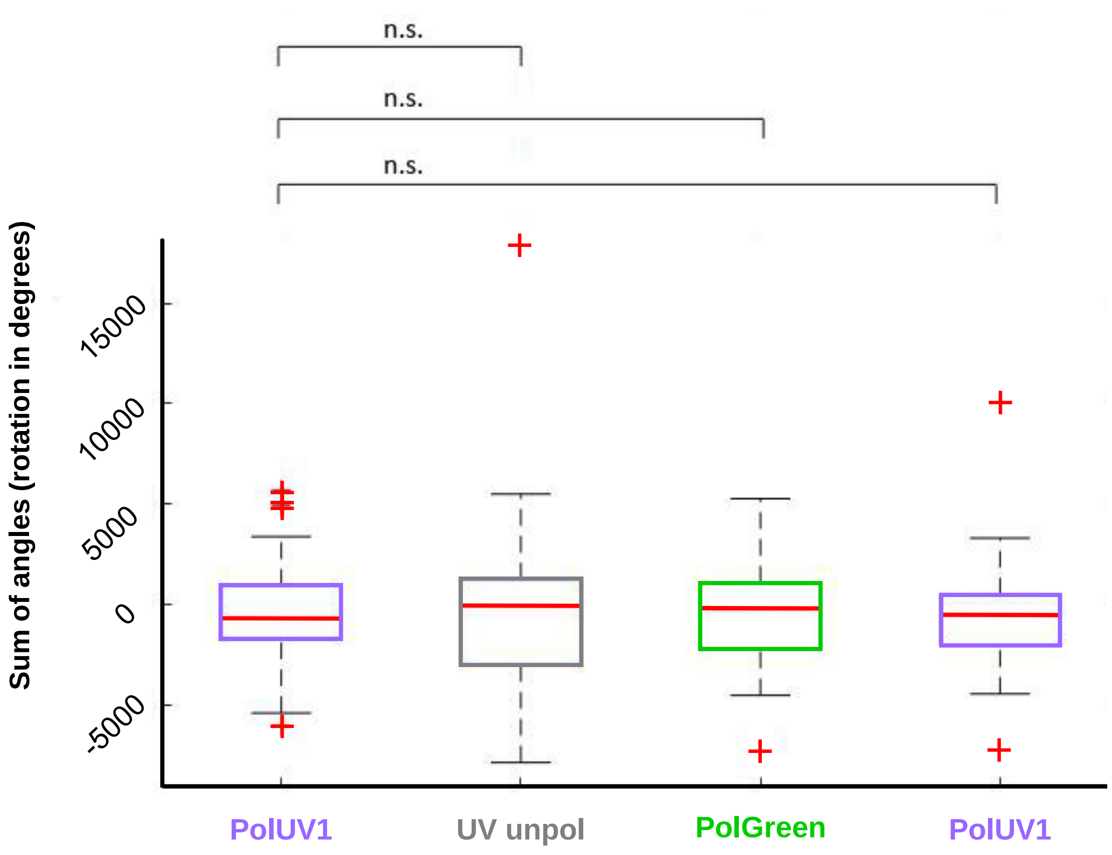

Supplemental Figure S4

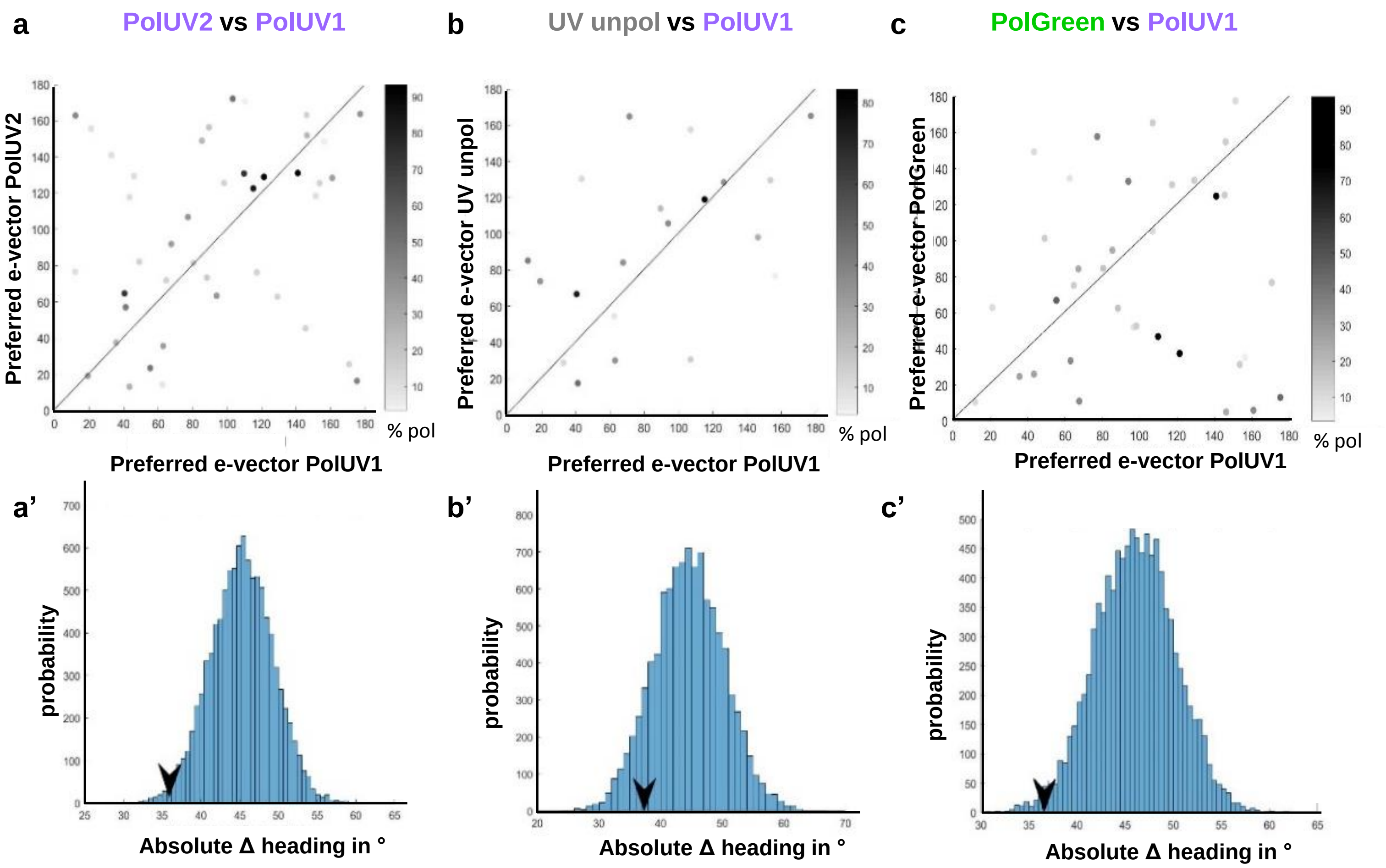

Supplement: Supplementary file 1 — Supplemental Figures and Legends [file 41598_2019_53330_MOESM1_ESM.pdf]
